# Kommerell’s diverticulum with right-sided aortic arch and anomalous origin of neck vessels: Uncommon imaging finding in neonate with cyanosis

**DOI:** 10.1259/bjrcr.20200173

**Published:** 2021-04-30

**Authors:** Darakhshan Kanwal, Safaa Khalil, Khaled Attia, Maged Fam, Mohammad Arakkal

**Affiliations:** 1 Department of Radiology, Al Qassimi Women and children Hospital, Sharjah, United Arab Emirates; 2 Department of Radiology, Al Kuwait Hospital, Sharjah, United Arab Emirates; 3 Department of Radiology, Al Qassimi Hospital, Sharjah, United Arab Emirates

## Abstract

Kommerell diverticulum is a rare developmental anomaly of aortic arch. It is most frequently seen with right-sided aortic arch and aberrant left subclavian artery or ligamentum arteriosum, which have a significant role in completing a vascular ring. However, aberrant origin of neck vessels along with it is not commonly seen. The signs and symptoms vary depending on the severity. The paediatric patients usually present early due to compression of mediastinal structures such as trachea or oesophagus.

We report a case of Kommerell diverticulum with right-sided aortic arch and anomalous origin of neck vessels which presented as recurrent apneic spells and choking attacks after feeding.

## Case details

### Clinical presentation

A 3-week-old neonate presented with history of apneic spell twice each lasting more than one minute associated with floppiness and bluish discoloration all over the body. Her mother gave history of recurrent choking attacks and vomiting after feeding since birth. The vomiting is non-projectile, nor blood or bile tinged. Her birth history was unremarkable (spontaneous vaginal delivery, birth weight 2.9 kg). No postnatal complications were noted. Currently, she is on combination of breastfeeding with formula feed.

On arrival, she was afebrile, heart rate 130/min, respiratory rate 42/min, SpO2 99% and weight 3.8 kg. On clinical examination she was alert, not in distress. All biochemical and haematological investigations were normal.

## Diagnostic workup

**Figure 1.**
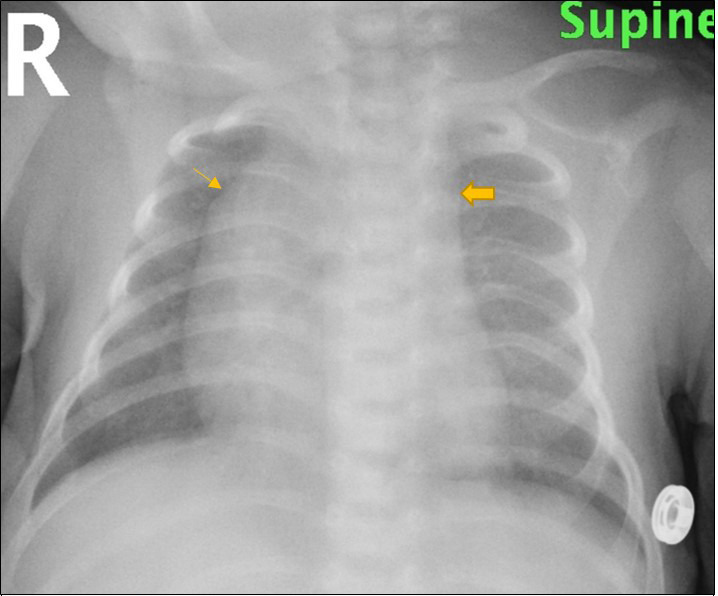
Chest X-ray frontal view shows straightening of the left cardiac border with loss of left aortic contour (block arrow) and prominent right para tracheal soft tissue (single arrow)

Chest X-ray in emergency department showed straightening of the left cardiac border with loss of left aortic contour and prominent right para tracheal soft tissue. (Figure 1)

Decision was made to admit the patient for close observation and workup for apneic episodes including echocardiography, upper GI contrast study and CT angiography of chest.

**Figure 2.**
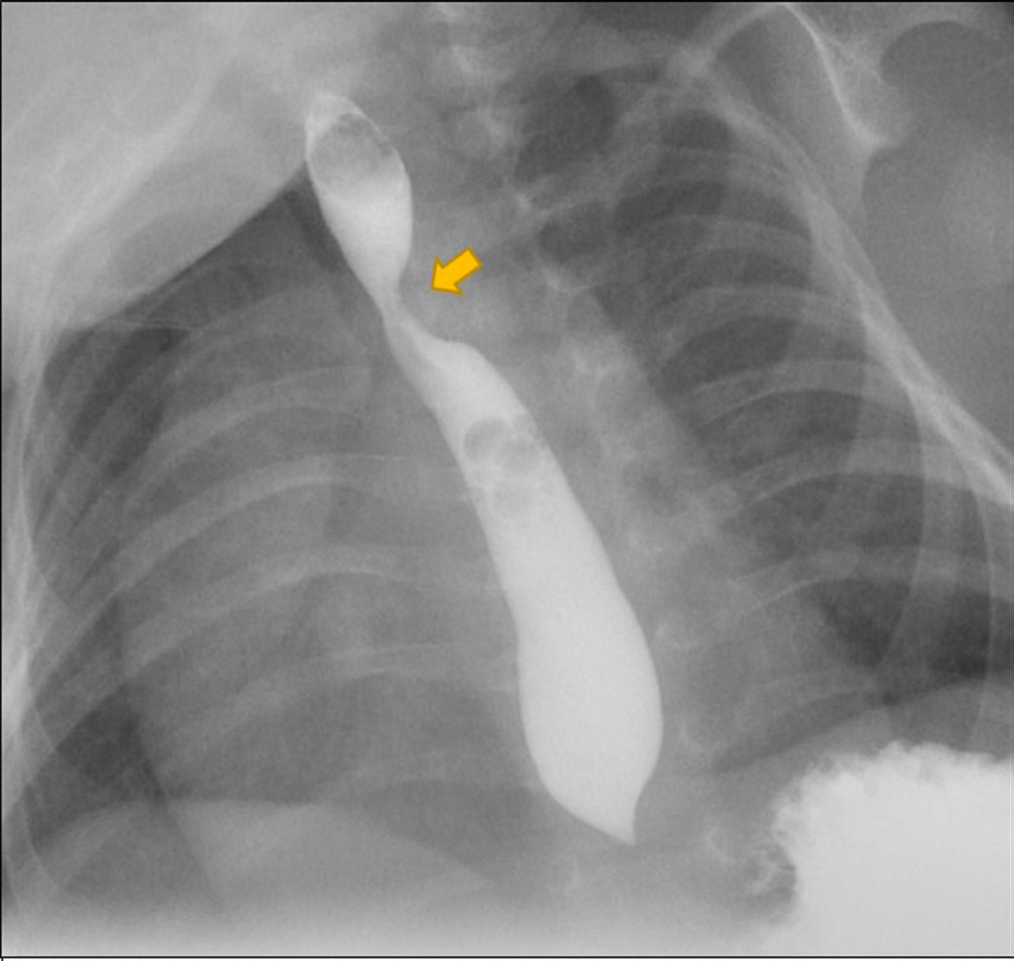
Upper GI contrast study (oblique view) shows postero-lateral indention on the contrast filled oesophagus at the level of 5th/6th thoracic vertebra. (block arrow)

Echocardiography showed normal left aortic arch with aberrant innominate artery from proximal descending aorta. Upper GI contrast study showed postero-lateral indention on the contrast column at the oesophagus just above the level of tracheal bifurcation opposite 5th/6th thoracic vertebra. However, normal distal flow of the contrast was noted (Figure 2)

**Figure 3.**
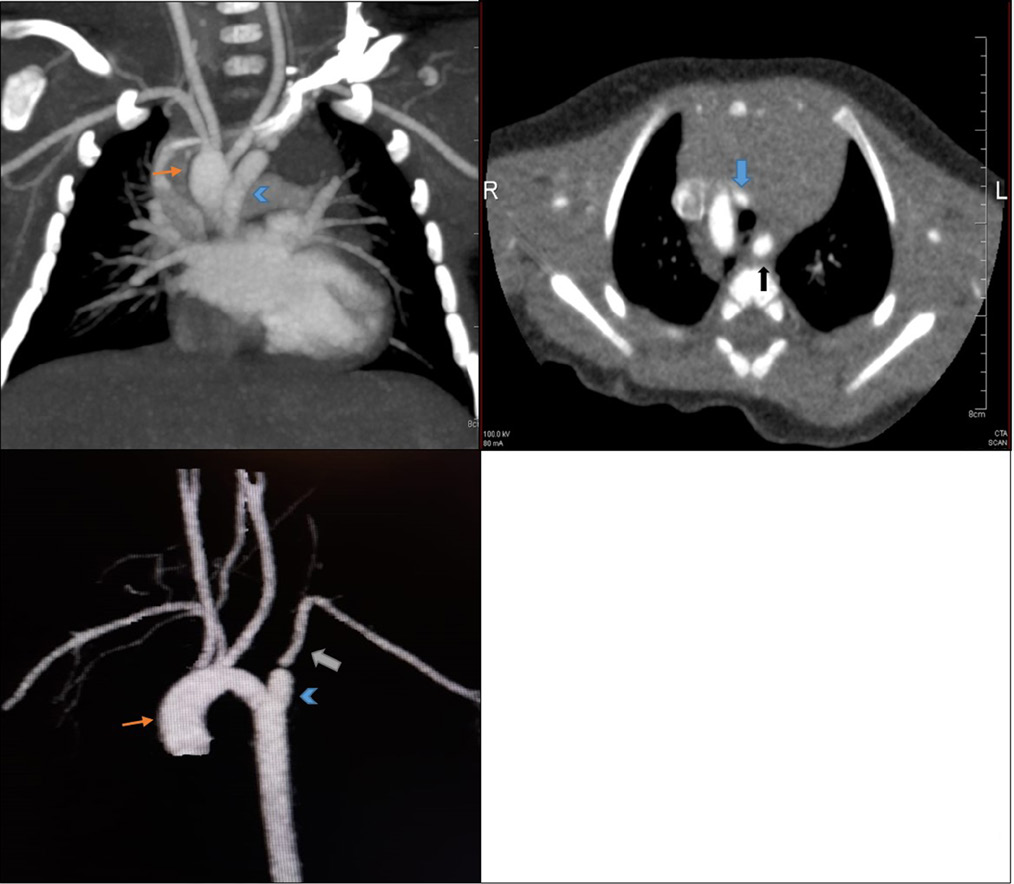
CT angiography (**a**) coronal, (**b**) axial and (**c**) 3D reconstructed views show right-sided aortic arch (orange arrow) with aberrant left subclavian artery ALSA (white arrow) which arises from a dilatation at its origin (KD; arrowhead). Left CCA arising from right-sided aortic arch coursing anterior to the trachea (blue arrow), ALSA courses posterior to oesophagus (black arrow) on the left side-making a vascular ring.

CT angiography revealed right-sided aortic arch with aberrant origin of left subclavian artery which arises from a dilatation at its origin (diverticulum of Kommerell) and significant narrowing of proximal part of the left subclavian artery. Left common carotid artery is arising from the right-sided aortic arch assuming a transverse oblique course anterior to the trachea. Aberrant left subclavian artery courses posterior to oesophagus seen on the left side-making a vascular ring. (Figure 3).

Preoperative flexible bronchoscopy and upper GI endoscopy was also performed to rule out any intraluminal pathology which were unremarkable.

## Management

Diagnosis was made of right-sided aortic arch with aberrant left subclavian artery arising from diverticulum of Kommerell and ligamentum arteriosum with tracheal and oesophageal compression. Surgery was planned and through left lateral thoracotomy division of ligamentum arteriosum, excision of Kommerell diverticulum, aortopexy and esophageal release was done.

Baby was stable after the surgery, stayed in ICU for close cardiorespiratory monitoring and discharged on 2nd post-operative day in stable condition.

## Discussion

Kommerell Diverticulum is the remnant of 4th dorsal aortic arch first described by Burckhard Kommerell, as aneurysmal dilatation of descending aorta at the origin of aberrant left subclavian artery (ALSA).^1,2^

Three types of aortic arch diverticulum have been described in literature.^3^

1. Aortic Diverticulum in Left Aortic Arch with Aberrant Right Subclavian Artery
2. Aortic Diverticulum in Right Aortic Arch with Aberrant Left Subclavian Artery
3. Aortic Diverticulum at the Aortoductal Junction

Based on Edwards classification, right-sided aortic arch can be further classified into three major subgroups^3^:

1. Type I includes right-sided aortic arch with mirror image arch branches.
2. Type II includes right-sided aortic arch with aberrant left subclavian artery and Kommerell diverticulum.
3. Type III includes right-sided aortic arch with isolated left subclavian artery communicating with the pulmonary artery.

Kommerell diverticulum (KD) with right-sided aortic arch (RAA) usually seen in 0.05–0.1% of the population.^4^ The left ligamentum arteriosum (LLA) joins the root of aberrant left subclavian artery (ALSA) to the left pulmonary artery (LPA).^5^ This along with RAA forms a vascular ring and typically seen with normal intracardiac anatomy.^6,7^ Our case was type II with significant stenosis at the origin of ALSA and aberrant origin of neck vessels from RAA. No intracardiac abnormalities were detected. Depending on severity, this vascular ring can cause symptoms of tracheal or oesophageal compression.^8^ In paediatric population, due to pliability of immature tracheal rings respiratory symptoms predominate.^9^ However in adults, common presentation is after the age of 40 years when aortic wall becomes atherosclerotic and rigid which can result in dysphagia, chest pain, cough and lower respiratory infection.^3^

Careful evaluation of aortic arch anatomy is crucial in planning for thoracic surgery and endovascular intervention, as well as to make decisions regarding repair of the coexisting lesions in the same procedure.^10^ At present, non-invasive modalities like echocardiography, cardiac magnetic resonance angiography (MRA) and CT angiography (CTA) has largely replaced invasive modalities like catheter angiograms in the diagnosis of vascular ring.^11^ In children, X-ray chest and echocardiography are considered as initial investigations in diagnosing congenital aortic arch anomalies.^12^ In patients presenting with dysphagia, contrast esophagography is performed while in children with respiratory symptoms bronchoscopy is used to rule out trauma or foreign body aspiration.^13^ However, evaluation by cross-sectional imaging for example CTA or MRA is mandatory in cases with complex anomalies, in older children and adults with inadequate or inconclusive echocardiographic findings.^14^

In assessing cardiovascular morphology, MRA has the advantage due to lack of ionizing radiation, use of small volume of non-iodinated contrast and evaluation of flow patterns like flow changes, pressure gradients etc.^15^ On the other hand, CTA is fast and provides 3D reconstructed images for illustrating the complex anatomical relationship between the aortic arch structures, trachea and oesophagus that helps in surgical planning.^16,17^ In our patient, after initial assessment CTA was planned as it was readily available and 3D images provided better understanding of vascular anatomy and its compression effects over the trachea and oesophagus. With the help of these imaging entities, KD can be diagnosed before lethal complications occur.^2^ Complications include aneurysm rupture, aortic dissection or recurrent pneumonia.^8^

Backer et al suggested that any diverticulum more than 1.5 times the diameter of subclavian artery should be considered for surgical intervention due to the increase likelihood of rupture or tracheoesophageal compression.^18^ Surgical treatment options include open repair, hybrid endovascular and total endovascular repair.^19^ In a study done by Shinkawa et al, it was found that excision of KD with interposition of left subclavian to left common carotid artery and division of ligamentum arteriosum have good prognosis in eliminating residual symptoms and late complications.^20^

## Learning points

1. Kommerell Diverticulum is a rare congenital anomaly which when occurs with right-sided aortic arch, aberrant left subclavian artery and ligamentum arteriosum forms a vascular ring.
2. Depending on severity, this vascular ring can cause symptoms of tracheal or oesophageal compression.
3. The role of imaging for evaluation of aortic arch anomalies and its associations is crucial for planning for surgery and endovascular intervention in symptomatic patients.
4. CT / MR angiography has gained more importance nowadays in non-invasive diagnosis of complex congenital anomalies.
5. If undiagnosed, lethal complications like aneurysm rupture and aortic dissection may occur.
